# Is Neurodevelopmental Assessment in Early Childhood Predictive of Performance Assessed Later in Childhood and Adolescence in Sub-Saharan Africa? A Systematic Review of the Literature

**DOI:** 10.1093/arclin/acad051

**Published:** 2023-07-19

**Authors:** Roméo Zoumenou, Florence Bodeau-Livinec, Léa Chausseboeuf, Michael J Boivin, Jaqueline Wendland

**Affiliations:** Institut de Recherche pour le Developpement, Mère et enfant face aux infections tropicales, 75006 Paris, France; Laboratoire psychopathologie et processus en santé, Institute de psychologie, 92774 Boulogne, France; Institut de recherche en santé, environnement et travail (IRSET), Ecole des hautes etudes en santé (EHESP), 93210 Saint-Denis, France; Laboratoire psychopathologie et processus en santé, Institute de psychologie, 92774 Boulogne, France; Department of Psychiatry and Department of Neurology & Ophthalmology, Michigan State University, East Lansing, MI 48824 USA; Department of Psychiatry, University of Michigan, Ann Arbor, MI 48104, USA; Laboratoire psychopathologie et processus en santé, Institute de psychologie, 92774 Boulogne, France

**Keywords:** Development, Cognitive ability, Assessment, Children, Africa, Review

## Abstract

**Background:**

Most neurodevelopmental tests used to assess child development in sub-Saharan Africa were developed in western or high-income countries, raising the question of their usefulness with African children.

**Objective:**

This systematic review identified and synthesized key findings from studies measuring development in children in Sub-Saharan Africa in early childhood and again at school age, to assess neurocognitive associations longitudinally from infancy through middle childhood.

**Methods:**

The study was based on the Preferred Reporting Items for Systematic Reviews and Meta-Analyses method, selecting articles referenced in the PubMed, PsycInfo, and Embase databases using the following inclusion criteria: published between 2000 and 2022, written in French or English, and presenting results dealing with the objective assessment of child’s neurodevelopment. All articles were registered in the Zotero reference manager and analyzed by title, abstract, and full text.

**Results:**

Several of the seven selected studies confirmed that attention and working memory in infancy can predict children’s neurocognitive performance, including mathematical ability, at school age. In two of the studies, children with poor mental development at 1 year of age are more likely to present with poorer behavioral development at school age, including learning difficulties in school and risk for grade repetition.

**Conclusion:**

Cognitive ability assessed in early childhood is strongly associated with performance at school age in cohorts of African children followed longitudinally. Even with assessments adapted cross-culturally, infants and preschoolers at risk for poor developmental outcomes can be identified to better receive strategic early interventions to enhance their development.

## Introduction

Child development is determined by both parental genes (heredity) and environmental conditions (Houdé, 2013: 21–36). Boivin and Giordani (2009) conducted a cross-cultural comparison of neuropsychological assessment studies of brain injury from comparable causes in sub-Saharan and in American school-age children (e.g., pediatric HIV, cerebral malaria versus sickle-cell disease, cognitive rehabilitation therapy for brain injury) (Boivin & Giordani, 2009). The consistency of findings for both African and American children when similar neuropsychological tests were used in such comparisons provided evidence for what Boivin and Giordani termed “a foundational brain-behavior omnibus.” The core idea is that this omnibus gives rise to various principal cognitive ability domains (e.g., attention, working memory, executive function, language) in a consistent manner for children globally, as sculpted by the socio-cultural environment. As each child is unique, one can observe differences from one child to another depending on their living conditions and environment. However, the foundational framework for the development of children in terms of their cognitive abilities as it unfolds throughout childhood is universal in terms of the principal domains and the kinds of assessments that can gauge an individual child’s cognitive performance in response to environments fostering both developmental risk and resilience (Boivin & Giordani, 2013).

In Sub-Saharan Africa (SSA), children are at a high risk of developmental delays and/or deficits as they accumulate many risk factors of an infectious, nutritional, and/or environmental nature (Walker et al., 2011). Within the neurocognitive domain, such deficits can encompass a child’s sensorimotor, language, social–emotional, and cognitive functions and abilities (Fourneret & Gentaz, 2022). Furthermore, many children face educational deficits in SSA, further undermining performance for these functions (Mingat et al., 2013). UNESCO in one of its reports (UNESCO, 2017), stressed the need for reforming and strengthening the basic education system to reduce illiteracy. In a series of cross-cultural studies of the correspondence between auditory/phonetic and visual–spatial working memory using the Kaufman Assessment Battery for Children (KABC), Boivin and colleagues observed that literacy was the single most important factor associated with the dominance of either of these working memory domains in terms of overall executive function. This was true for Congolese (Conant et al., 1999), Lao (Conant et al., 2003), and for Senegalese and Ugandan school-age children (Boivin et al., 2010a).

Access to education is difficult in some regions of the sub-Sahara due to poverty and ethnic and political rivalries; school infrastructure is lacking, and that which does exist is undermined by civil, ethnic, and political instability. In addition, parents with little or no schooling are unable to support their children in their educational progression, often causing parents to prioritize educational opportunities for their children on the basis of gender and age. However, when children receive quality care and education in early childhood, they achieve fundamental milestones in their cognitive and emotional development (Boivin et al., 1996; Boivin et al., 2013a; Boivin et al., 2013b; Boivin et al., 2017a; Boivin & Davidson, 2018). As much as 66% of children under the age of five fail to reach their full developmental potential (Drago et al., 2020), resulting in lower individual and national productivity (Black et al., 2017; Ribe et al., 2018).

Ugandan children, however, at risk from perinatal infection and/or exposure to maternal HIV can see significant developmental gains in their preschool years when their mothers receive biweekly training on practical strategies to enrich their children’s emotional, social, and cognitive skills in daily parent/child interactions in the home (Bass et al., 2016; Bass et al., 2017; Boivin et al., 2013a; Boivin et al., 2013b; Boivin et al., 2017a). These caregiver training benefits can extend to the older school-age siblings in the household, as witnessed by improved KABC overall cognitive performance for nontarget siblings in the intervention households (Boivin et al., 2020a). However, data on the neurodevelopmental risk of children in developing countries are often based on indicators of poverty and malnutrition and rarely on the actual developmental assessment of the affected children (Black et al., 2017; Black et al., 2023). Such indicators are limited when used as proxies for a population’s child development risk, and several recent studies have advised that assessments be administered directly to the children and performance based (Ogunrin & Adamolekun, 2007; Scharf et al., 2018; Zuilkowski et al., 2016).

Neuropsychological assessment methods have progressed beyond screening-based observations of developmental milestones that rely only on parental memory or pediatric clinical impressions (Ogunrin & Adamolekun, 2007). For developmental science to progress in the global context, physician/scientists need to develop assessments that are culturally adaptable, valid, reliable, and feasible in low- and middle-income country (LMIC) settings (Abubakar et al., 2010; Holding et al., 2018). When assessing child development in the African context, investigators also need a list of assessment resources along with guidance on how best to adapt western-based developmental assessments in culturally appropriate and valid ways for research and clinical use for a given type of medical or social disability assessment use (Semrud-Clikeman et al., 2017). For example, the Malawi Developmental Assessment Tool (MDAT) (Gladstone et al., 2010) and the Object-based Pattern Reasoning Assessment (OPRA) (Zuilkowski et al., 2016) use materials that African children easily recognize. Likewise, the Kilifi Developmental Inventory (KDI) for early childhood (Abubakar et al., 2008; Abubakar et al., 2010), and the Kilifi Developmental Checklist (KDC) for school-age children (Holding et al., 2018; Penny Holding & Boivin, 2013), have undergone extensive validation in the African context for research and clinical use, especially for Kenyan children surviving severe malaria (Holding et al., 1999; Holding et al., 2004; Holding & Kitsao-Wekulo, 2004; Holding & Snow, 2001).

Computer-based cognitive training games designed using an African village motif has also been shown to be effective in improving neurocognitive performance in Ugandan children living with HIV (Giordani et al., 2015; Novak et al., 2017). However, more culturally neutral nonverbal computerized cognitive games, such as those based on playing cards (e.g., CogState), can also be effective in the neuropsychological assessment of school-age African children (Bangirana et al., 2015). Likewise, culturally neutral computer cognitive games tasks that are not language dependent can be effectively used to assess learning abilities with both school-age Ugandan children surviving severe malaria (Bangirana et al., 2011; Bangirana et al., 2013; Bangirana et al., 2009), and living with HIV (Boivin et al., 2010b; Boivin et al., 2019b).

## Deficit of psychometric tests in SSA

The paucity of developed and standardized tests in SSA has led researchers and professionals to use those developed and calibrated in high-income Western countries to assess children’s development (Bodeau-Livinec et al., 2019). This raises issues regarding the administration and interpretation of results in different cultural contexts (Holding et al., 2004; Holding et al., 2018). SSA-population-based normative data with reference to these Western tests are generally unavailable; thus, a variety of statistical strategies are used to standardize test scores on the basis of age or gender (Bergemann et al., 2012; Bodeau-Livinec et al., 2019). Furthermore, the validity of the tests at the clinical level has been questioned (Kammerer et al., 2013). However, despite the interest among researchers, professionals, clinicians, and policymakers in assessing child development, relatively little work has been conducted to develop appropriate local measures (Zuilkowski et al., 2016). As early neurocognitive and social–emotional development are robust determinants of children’s academic progress in developed countries (Grantham-McGregor, 2016; Grantham-McGregor et al., 2007), establishing whether developmental measures in childhood are reliable predictors of later development in SSA children is important (Kammerer et al., 2013).

A final consideration in this review is whether we should compare effect sizes for studies with African children with American or western studies to address the validity, appropriateness, and/or error of Western measures used in the SSA or other LMIC cultural settings. One example of this type of analysis was completed by Holding and Boivin in their review of the assessment of neuropsychological outcomes in pediatric severe malaria studies in the sub-Sahara (Holding & Boivin, 2013). When comparing cerebral malaria survivors when non-malaria controls for persisting developmental and neurocognitive disabilities, Holding and Boivin presented detailed tables of effect sizes (Cohen’s *d* statistic) for such comparisons using western-based neuropsychological tests for Ghana (Dugbartey, 1995; Dugbartey et al., 1998a; Dugbartey et al., 1998b; Dugbartey & Spellacy, 1997) and Gambia (Muntendam et al., 1996); adaptations of such tests for the local cultural context after in-depth validation for Kilifi Kenya (Holding et al., 1999; Holding et al., 2004), and again with a more extensive cognitive and language assessment battery adapted for the sub-Saharan context (Carter, 2002; Carter et al., 2003a, 2003b, 2004, 2005a, 2005b, 2006). The review by Holding and Boivin also included detailed effect sizes for pediatric cerebral malaria studies that used western-based cognitive assessment battery comprised of the Tactual Performance Test (TPT), the KABC, and the Tests of Variables of Attention (TOVA) in a retrospective study of Senegalese CM survivors (Boivin, 2002), as well as in prospective studies of CM survivors in Uganda (Bangirana et al., 2011; Boivin et al., 2007; John et al., 2008). Finally, consistent findings were observed in a review of the neurodevelopmental effects of early cerebral malaria exposure included a prospective study using the Malawi Developmental Assessment Test (MDAT). This corroborative supporting evidence to CM neurocognitive outcomes in Uganda and Senegal using Western-based tests is important, as the MDAT was developed, validated, and normed in Malawi especially for the sub-Saharan cultural context (Boivin et al., 2011).

Holding and Boivin concluded from their review that significant effects sizes for deficits could be observed for some, if not most, of the developmental, neuropsychological, and neurocognitive domains evaluated for the CM survivors in comparison to their non-malaria controls. The only exception was Muntendam et al.’s (1996) study in Gambia, where no differences were observed for the CM and control children. However, the lack of significant CM/control cohort neurocognitive differences in this study was perhaps because of the exclusion of CM cases with any neurological signs during acute illness or afterwards. Even though significant effect sizes could be observed across all the different sub-Saharan country sites reviewed for CM survivors, the multiplicity of tests and their cultural origins made it difficult to arrive at a robust neuropathological developmental profile for survivors of this disease. It was only in a systematic analysis across the various studies which all assessed a broad range of developmental and neurocognitive domains that a general profile of the neuropsychological sequelae of CM could be deciphered. This was true irrespective or the extent to which a test originated from a high-income country (HIC) or not (Holding et al., 2018; Holding & Boivin, 2013).

However, the more extensive the adaptation and validation work for a given test battery for the sub-Saharan context, the more consistent the significance of all of the principal neurocognitive domain effect sizes (Holding et al., 2018). When resources are not available for such extensive adaptation and validations, an HIC-based assessment battery appropriately applied in a study design that includes matched comparison groups within that cultural context. When this is the case, HIC-based tests can lead to robust developmental findings in terms of identifying the neurocognitive and developmental deficits in African children living with brain injury from infectious disease (Boivin et al., 2018, 2020b). Again, Boivin and colleagues have hypothesized that this is because of a foundational brain-behavior omnibus undergirding human development across the lifespan for the human species (Boivin et al., 2013c; Boivin & Davidson, 2018; Boivin & Giordani, 2009).

A scientific analysis supporting the utility of HIC-based tests for the sub-Saharan context was well documented in a systematic review of cross-cultural assessment of HIV-associated cognitive impairment using the KABC (van Wyhe et al., 2017). Through the use of relevant keywords and MeSH terms entered into PubMed, PsychInfo, EBSCOHost, ProQuest, and Scopus databases — van Wyhe and colleagues were able to identify nine studies in which the KABC was used to compare neurocognitive profiles of HIV-infected, exposed but not infected, and non-exposed/non-infected children. Eight of these studies were in African countries (five in Uganda, two in South Africa, and one multi-site study with children from Malawi, Uganda, and Zimbabwe). The remaining study was done in the United Kingdom. Even though the KABC was adapted directed to such a wide range of SSA study locations, langages, and nationalities, consistent neurocognitive deficit profiles emerged for the HIV-infected children. This was especially the case for the KABC Simultaneous Processing (visual–spatial analysis and problem solving) and working memory (Sequential Processing) domains. This was the case despite the anti-retroviral treatment (ART) status and clinical stability of the samples of infected children (van Wyhe et al., 2017). This was perhaps the most rigorous evaluation to date of the suitability of an HIC-based test (i.e., the KABC) for use in providing neurocognitive outcomes for studies of a given brain injury exposure (perinatal HIV) in a wide range of cultural contexts in both SSA and the UK.

Likewise, in the present review, all of the studies included became part of an extensive systematic review (Preferred Reporting Items for Systematic Reviews and Meta-Analyses, PRISMA guidelines) focused on the application of HIC-based tests (e.g., KABC/KABC-2); as applied to the study of one disease conditions (e.g., pediatric HIV); and including only studies with appropriate methodological comparison groups (e.g., comparison groups of non-infected and or non-infected and non-exposed controls). Also, this review included on studies analytically designed to assess developmental outcomes after adjusting for appropriate analytical controls (e.g., SES, gender, education level and schooling, nutritional status, ARV treatment status, clinical stability as covariates). We anticipate that the present review findings will provide further support for a foundational brain/behavior omnibus at the core of a useful paradigm supporting child developmental assessment science (Boivin & Giordani, 2009, 2013). Towards that end, we propose the following primary object for the present systematic review.

## The primary objective of this review

The primary objective of this review is to examine the association between outcomes from the assessment of neurocognitive development in childhood, and school-age outcomes assessed at later points in children and adolescents in SSA. We will identify and synthesize key findings from studies that have measured neurocognitive development in cohorts of SSA children at a minimum of two different ages to assess whether developmental measurements in early childhood are associated with later neurocognitive, social-emotional development, or academic outcomes.

## Methods

### Design and data selection

In conducting a systematic review of the literature, we referred to the standards of the PRISMA statement (Moher et al., 2009). We formulated our research question using the Population, Intervention, Comparison, and Outcome method as follows (Huang et al., 2006): Is the measurement of children’s neurocognitive development associated with their later development (cognitive, social–emotional, and motor development and academic outcomes)?

### Search strategy

**Fig. 1:**
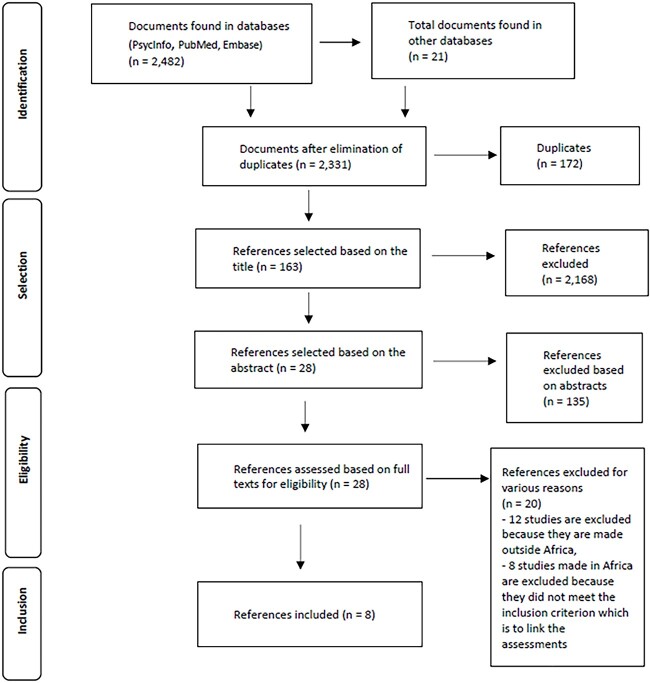
PRISMA Flow diagram for identification of studies from databases and registers.

We collected articles between January and February 2022, with the final update taking place in July 2022. References were selected from the PubMed, PsycInfo, and Embase databases, and an additional manual search was performed on Google Scholar. After storing the search results in the reference manager Zotero, we selected appropriate articles using the following combination of keywords: (Africa OR sub-Saharan Africa) AND (neuro* OR 5cogniti* OR development*) AND (child* OR kid* OR children*) AND (cohort). After applying the search string to the databases, the duplicated results were removed. The remaining articles were then examined by title and abstract for compliance with the selection criteria, and finally, full-text analysis was performed to verify if they met our inclusion criteria. The full-text articles were independently reviewed by two researchers, and disagreements were examined in detail to reach a consensus. The data collection process is presented in the flow chart in Fig. 1.

### Selection criteria

#### Eligibility criteria

To meet the eligibility criteria, we selected references addressing the objective assessment of child development in SSA and presenting results predictive of child development. The studies were required to include at least two measures of child/adolescent development; involve a qualitative, quantitative, or mixed-methods approach; and be published between 2000 and 2022. The following data were extracted from the included studies: the country of study, study objective, target population, age of children/adolescents, health problems of the study population, method of assessment, and main outcomes (Table 1).

**Table 1:** Selection of articles

Authors, title, and year of publication	Country of study	Objective and design of the study	Study population (age and/or sex)	Health problems of the study population	Method/Evaluation (types of tests used)	Number of interventions and evaluation period	Key results
**Boivin et al.,** Neurodevelopmental assessment at 1 year of age predicts neuropsychological performance at six years in a cohort of West African children (2021)	Benin	A longitudinal study linking a neurodevelopmental assessment based on Western methods in early childhood (1 year) and neuropsychological performance at school age (6 years)	*N* = 568, with 521 6-year-old children who completed the tests included in the analysis: 251 girls and 270 boys	No health problems.	In this study, children were assessed on their psychomotor development with the MSEL at 1 year and on their neurocognitive development with the KABC-II, BOT2, and TOVA at 6 years. The children’s mothers were assessed with the EPDS, Raven, and Caldwell HOME to investigate their home environments.	Two interventions: at 1 and 6 years	MSEL composite and gross motor scores were statistically significant predictors of KABC-II and BOT-2 scores but not of TOVA scores. Cross-culturally-adapted early childhood neurodevelopmental assessment predicts a child’s cognitive and neuropsychological abilities at preschool age.
**Gandhi et al.,** Child development at 5 years of age predicted mathematics ability and schooling outcomes in Malawian adolescents (2012)	Malawi	A prospective cohort study examining the association between child development at age 5 and mathematical ability and school achievement at age 12 in children in Malawi	*N* = 813 children, of whom 415 were included in the analysis at 5 and 12 years	The assessed children come from a cohort assessed at 5 years and subsequently 12 years of age and have no health problems.	This study involved an assessment of development at 5 years of age based on the Denver II, Denver Developmental Screening Test, and Griffiths Mental Developmental Scales (this assessment concerns gross and fine motor skills, language, and socialization). Subsequently, at 12 years of age, each child was evaluated on their health, anthropometry, academic performance, and their passing of a mathematics test.	Two interventions: at 5 and 12 years	The results are based on assessments at age 12: the percentage of correct answers given to mathematics test questions, the highest grade level attained, and the number of grade repetitions. Child development at age 5 showed signs of a positive association with mathematical abilities and perhaps the highest grade level reached at age 12.
**Cortina et al.,** Relationship between children’s cognitions and later educational progress in rural South Africa: a longitudinal study (2019)	South Africa	A longitudinal study examining the effects of children’s mental health and cognitions on educational outcomes in low- and middle-income countries (LMICs)	A total of 1025 children aged 10 to 12 years participated in the evaluation, and, 5 years later, 528 (51.5%) of these children could be located and linked to population data to assess educational outcomes at approximately 16–18 years.	No health problems.	The Strengths and Difficulties Questionnaire (SDQ) and Cognitive Triad Inventory for Children (CTI-C) were used to assess the psychological and cognitive functioning of children between the ages of 10 and 12. These data are linked to measures of academic progress collected 5 years later by examining associations between educational progress and behavioral and emotional problems and cognitive interpretations.	Two interventions: between 10 and 12 years, and between 16 and 18 years	The results of this study suggest that if a child can develop a positive view of themselves, the future, and the world around them, they are more likely to achieve better educational progress. Interventions that reinforce the cognitive style of childhood have the potential to reduce early school leaving and increase educational attainment in LMICs, thereby improving trajectories throughout life. Cognitive style, therefore, predicts educational progress in this study.
**Hsiao & Richter,** Early mental development as a predictor of preschool cognitive and behavioral development in South Africa: the moderating role of maternal education in the Birth to Twenty Cohort (2014)	South Africa	An article examining the influence of early development on preschool cognitive and behavioral outcomes in South Africa and the role of family factors, such as maternal education, in moderating this association	The study involved 167 Black South African children, including 89 boys and 78 girls, from the Birth to Twenty Cohort during their first 5 years of development.	No health problems.	The following tests were used: the Griffiths Mental Development Scales (GMDS) for 1-year-olds, and the Denver Prescreening Developmental Questionnaire (PDQ) and the Revised Denver Prescreening Developmental Questionnaire (R-PDQ) for 5-year-olds. The following aspects of development were assessed: social-staff, gross and fine motor skills, language, and cognitive abilities.	Two interventions: at 1 and 5 years	The results indicate that mental development assessed at age 1 can significantly predict preschool outcomes at age 5, beyond the contribution of maternal education. Children with lower mental development at age 1 also displayed the lowest cognitive and behavioral development at age 5. Higher levels of maternal education mitigated the negative impacts of early developmental delay on preschool cognitive and behavioral outcomes. Maternal education, thus, compensates for the poor development of infants.
**Richter et al.,** Predictive power of psychometric assessments to identify young learners in need of early intervention: data from the Birth to Twenty Plus Cohort, South Africa (2015)	South Africa	A study assessing the predictive power of eight psychometric assessments administered during infancy as screening measures to identify individuals in need of early interventions to prevent late school entry and grade repetition	This analysis used data generated as part of the Birth to Twenty Plus (Bt20+) study, an ongoing longitudinal birth cohort project that examines the socioeconomic aspects, growth, health, development, and overall well-being of urban South African children and their families. In the study, 3273 individuals comprising only children were recruited during this period, and approximately 70% of the children have been monitored for 25 years to date.	No health problems.	The measures used were the Bayley Scales of Infant Development and the Griffiths Mental Development Scales at 6 months and 1 year of age; the Vineland Social Maturity Scale and the behavioral screening questionnaire at 2 and 4 years; the Revised Denver 5-Year Screening Development Questionnaire; and Conners’ Teacher Rating Scale, Draw-a-Person test, and Raven’s colorful progressive matrices at age 7. The analysis of the characteristic curve of receiver operation was used to examine the predictive values of the measurements, and the area under the curve was used to assess sensitivity and specificity.	Six interventions: at 6 months and 1, 2, 4, 5, and 7 years	The results suggest that with a moderate degree of diagnostic accuracy, the Bayley Scales of Infant Development at 1 year of age with a characteristic curve of receptor functioning and Conners’ Teacher Rating Scale at age 7 with a characteristic curve of receptor functioning can be used as screening measures to identify children at risk of delayed school entry. Conners’ Grade 7 Teacher Rating Scale predicted grade repetition with moderate accuracy. The only statistically significant covariate-adjusted model showed that a young maternal age and low socioeconomic status had a negative influence on the age of school entry, as predicted by the Bayley Scales of Infant Development in grade 1.
**Familiar et al.,** Early Childhood Vigilance Test (ECVT) of Attention in Younger HIV-Exposed Ugandan Children Predicts Tests of Variables of Attention (TOVA) at School Age (2022)	Uganda	A study aiming to assess whether Early Childhood Vigilance Test (ECVT) results in HIV-exposed/uninfected preschool children predicted their neurocognitive performance at school age using two neuropsychological measures: TOVA and KABC-II	Of the 44 children assessed with the ECVT, the study reassessed 38 school-age HIV-uninfected children, including 17 boys and 21 girls, with the TOVA and KABC-II tests.	The children were exposed to HIV but not infected.	The tests used were the ECVT, HOME, TOVA, and KABC-II. Two measurements were carried out between 3 and 5 years and then between 5 and 9 years. The children were assessed with the ECVT and HOME between 3 and 5 years and the TOVA and KABC-II between 5 and 9 years.	Two interventions: between 3 and 5 years, and between 5 and 9 years	Measuring attention in early childhood predicts computational measures of attention at school age and can be used to assess the effects of early risk and resilience factors on brain and behavioral development in African children affected by HIV.
**Familiar-Lopez et al.,** Predictive validation of Ugandan infant eye-tracking test for memory of human faces (2022)	Uganda	A study aiming to show that an eye-tracking measure of infant attention and working memory can predict aspects of neurocognitive performance in later years in Ugandan children	A total of 62 HIV-exposed uninfected children were assessed at enrolment (6 to 12 months) with the Mullen Early Learning Scales and the Fagan Test for Infant Intelligence (FTII). Of these children, 49 were reassessed at follow-up visits with the KABC-II and TOVA tests at preschool age (3–5 years).	The children were exposed to HIV but not infected.	Between 6 and 12 months, the children were assessed using the MSEL and FTII tests. During follow-up visits, these same children were reassessed with the KABC-II and TOVA at preschool age.	Two interventions: between 6 and 12 months, and between 3 and 5 years	The eye-tracking measurement of infant attention and working memory can predict aspects of neurocognitive performance in later years in Ugandan children. Cognitive assessments adapted to eye-tracking instrumentation may be useful for assessing attention and working memory in HIV-positive children living in LMICs.

#### Exclusion criteria

The following articles were excluded: systematic reviews, meta-analyses, or exclusively theoretical articles; those presenting test validations; and those involving methodology that did not comply with the selection criteria. Studies conducted outside of SSA were also excluded (see Table 2).

**Table 2:** Exclusion criteria

**Authors and year**	**Study title**	**Reason for exclusion**
**Van Schie et al.,** 2010	Motor testing at 1 year improves the prediction of motor and mental outcome at 2 years after perinatal hypoxic–ischaemic encephalopathy	The studies took place in the Netherlands on a small sample of 32 children.
**O'shea et al.,** 2018	Accuracy of the Bayley-II mental development index at 2 years as a predictor of cognitive impairment at school age among children born extremely preterm	The study is not carried out in sub-Saharan Africa but in the United States.
**Domsch et al.,** 2009	Prediction of childhood cognitive abilities from a set of early indicators of information processing capabilities	The study was carried out in Germany, so the socio-economic realities are different from Africa.
**Charkaluk et al.,** 2017	Ages and Stages Questionnaire at 3 Years for Predicting IQ at 5–6 Years	The EDEN mother–child cohort study on which this article is based was carried out in the university maternity hospitals of Poitiers and Nancy in France
**Erdei et al.,** 2020	Predicting School-Aged Cognitive Impairment in Children Born Very Preterm	Study carried out in New Zealand
**Rochat et al.,** 2018	Prevalence and risk factors for child mental disorders in a population-based cohort of HIV-exposed and unexposed African children aged 7–11 years	The study examined the relationship between the mother and childhood mental disorders. So does not correspond to the objectives of the research.
**Lodha et al.,** 2009	Clinical Risk Index for Babies score for the prediction of neurodevelopmental outcomes at 3 years of age in infants of very low birthweight	This study took place in Canada with the Clinical Risk Index for Babies-revised (CRIBII) test, which is a less used test in Africa.
**Janssen et al.,** 2009	A model to predict motor performance in preterm infants at 5 years.	The study is carried out in the Netherlands.
**Ajayi et al.,** 2017	Factors Associated with the Health and Cognition of 6–8-Year-old Children in KwaZulu-Natal, South Africa	The methodology does not correspond to the objectives of this study. The aim here is to study the link between the health, nutritional status and cognitive development of children aged 6 to 8.
**Greene et al.,** 2012	Evaluating preterm infants with the Bayley-III: Patterns and correlates of development	The study took place in the United States, not Africa.
**Hohm et al.,** 2007	Language development at ten months: Predictive of language outcome and school achievement ten years later?	Reject because, this is a study carried out in Germany.
**Claas et al.,** 2010	Neurodevelopmental outcome over time of preterm born children ≤750 g at birth	The study was done in the Netherlands.
**Boivin et al.,** 2019	Neurodevelopmental effects of antepartum and postpartum antiretroviral exposure for HIV-exposed versus HIV-unexposed uninfected children in Uganda and Malawi: a prospective cohort study	Reject because the method used does not predict the cognitive development of the child.
**Zuilkowski et al.,** 2016	Dimensionality and the Development of Cognitive Assessments for Children in Sub-Saharan Africa	The methodology does not correspond to the objectives of this study
**Koura et al.,** 2013	Usefulness of child development assessments for low-resource settings in francophone Africa	The study is cross-sectional, and the methodology does not respect the predictive aspect.
**Rimvall et al.,** 2014	Predicting ADHD in school age when using the Strengths and Difficulties Questionnaire in preschool age: a longitudinal general population study, CCC2000	Reject because the study was carried out in Denmark
**Simard et al.,** 2011	Prediction of developmental performance in preterm infants at two years of corrected age: Contribution of the neurological assessment at term age	This is a study conducted in Montreal, Canada.
**Boivin,** 2002	Effects of Early Cerebral Malaria on Cognitive Ability in Senegalese Children	Although the study takes place in Senegal the method did not respect the predictive nature of the test
**Gladstone et al.,** 2010	The Malawi Developmental Assessment Tool (MDAT) The Creation, Validation, and Reliability of a Tool to Assess Child Development in Rural African Settings	Creating a Child Development Assessment Tool: Validation of a Test.
**Casale et al.,** 2014	The association between stunting and psychosocial development among preschool children: a study using the South African Birth to Twenty cohort data	Although conducted in South Africa, this study examined the association between stunting and psychosocial development.

## Results

All the identified studies are quantitative and used a longitudinal design. After running the search string on the databases, 2482 articles were extracted; in addition, 21 articles were included after a manual search on Google Scholar. After the removal of duplicates, the resultant 2331 articles were organized by title, from which a total of 163 articles were retained. Subsequently, 28 articles were selected from this total based on their abstracts and then evaluated according to their full texts. We mainly excluded tool validation studies and those with methodologies that did not comply with the selection criteria. Of the 29 full-text reviewed articles, only 7 met the inclusion criteria. The seven selected studies were from South Africa (three), Uganda (two), Benin (one), and Malawi (one). Twelve studies were excluded because they were conducted outside of SSA (in Western countries), and the remaining eight, although conducted in SSA, did not meet the inclusion criteria and were also excluded (see Table 2).

### Objectives and design of the studies

Developmental neuropsychology combines developmental psychology with neuroscience to study brain/behavior functions throughout the life span, but especially in childhood and adolescence. Brain/behavior function as derived by genetic, environmental, and epigenetic influences is foundational to a child’s cognitive, motor, behavioral, and psychosocial adaptation in daily life (Boivin et al., 2013d). Because of this broad and inclusive approach to assessment of child development, predicting neurocognitive development from objective measures during childhood was not consistently the primary objective of the included studies but was one of the important parameters investigated (Busman et al., 2013a, 2013b). Our review also encompassed relevant cross-cultural studies of behavior and psychosocial adjustment, as well as how these might be related to academic skills for the child (Bangirana et al., 2011). Therefore, the objectives of the present systematic review included studies evaluating:

- neurocognitive development and neuropsychological performance at school age (Boivin et al., 2021);
- child development and academic progress (Gandhi et al., 2013);
- the effects of mental health and cognition on school outcomes (Cortina et al., 2019);
- the influence of early development on cognitive and behavioral outcomes (Hsiao & Richter, 2014);
- psychometric assessments and identification of individuals in need of interventions (Richter et al., 2015); and
- the assessment of alertness, attention, and neurocognitive performance (Familiar et al., 2022; Familiar-Lopez et al., 2023).

### Study target populations

The systematic review included all studies that have assessed children of less than 17 years of age from both sexes at least twice in the sub-Sahara. The studies mainly took place in rural areas in developing SSA LMICs. The children assessed in the selected studies have no identified serious health problems and are individuals at risk mainly because of their socioeconomic status (SES).

### Evaluation methods

The included studies address children’s development and well-being using objective measures of development. Several neurocognitive developmental tests were used for the assessments. Three studies used the Kaufman Assessment Battery for Children, second edition (KABC-II) and the TOVA (Boivin et al., 2021; Familiar et al., 2022; Familiar-Lopez et al., 2023) to assess neuropsychological development in children. Two studies used the Revised Denver Prescreening Developmental Questionnaire (R-DPDQ) to assess children’s cognitive functions (Hsiao & Richter, 2014; Richter et al., 2015); and two employed the simple Denver II form (Gandhi et al., 2013; Hsiao & Richter, 2014). The Griffiths Mental Development Scales (GMDS) were used in three studies to assess motor skills (Gandhi et al., 2013; Hsiao & Richter, 2014; Richter et al., 2015). One study employed the Bayley Scales of Infant Development (BSID) and the Vineland Social Maturity Scale (VSMS) to study children’s social maturity (Richter et al., 2015). The Mullen Scales of Early Learning (MSEL) were used in two studies to assess children’s psychomotor development (Boivin et al., 2021; Familiar et al., 2022); and the Strengths and Difficulties Questionnaire (SDQ) was used in two studies for emotional and behavioral screening (Boivin et al., 2021; Cortina et al., 2019). The Cognitive Triad Inventory for Children (CTI-C) was used in one study to assess children’s psychological functioning (Cortina et al., 2019). The South African Child Assessment Schedule, the Youth Self-Report Questionnaire, and the World Health Organization (WHO) Self-Report Questionnaire were used to determine externalizing disorders in children (Orri et al., 2022). Similarly, the Early Childhood Vigilance Test (ECVT) was used in one study and the Fagan Test for Infant Intelligence (FTII) in another to measure the early childhood attention and vigilance (Familiar et al., 2022; Familiar-Lopez et al., 2023).

### Main findings

All the included studies observed a relationship between children’s neurocognitive assessment and their later neurocognitive, behavioral, social-educational, and/or academic development (Table 1). The following results can be highlighted:

#### Predictive power of neurocognitive development

One of the selected studies addressed the prediction of neuropsychological performance based on neurocognitive measures of children, confirming that neurodevelopmental assessment at one year of age can predict children’s neurocognitive performance at 6 years (Boivin et al., 2021). Different neurodevelopmental assessments are predictive of children’s cognitive performance in later years. The MSEL cognitive composite total at 1 year of age was significantly predictive of all the KABC-II cognitive performance domains at 6 years of age (Sequential Processing (*p* < 0.001), Simultaneous Processing (*p* = 0.044), Learning (*p* = 0.224), Delayed Recall (*p* = 0.016) with the partial eta squared value ranging from 0.19 (Sequential Processing) to 0.13 (Delayed Recall). All the partial eta values (after adjusting for maternal caregiving quality, depression, home environment, and maternal literacy and cognitive ability) were in the moderate to strong category in terms of clinical power.

The MSEL cognitive composite was not significantly predictive for any of the primary performance outcomes for the TOVA, including signal detection performance, percent omission error to signal presentation, percent commission errors to non-signal presentation, response time variability for responses to signal, and speed of response time to signal. Likewise, none of the partial eta-squared values were beyond weak when adjusting for all the model covariates. MSEL gross motor development at 1 year was significantly predictive of the Bruininks-Oseretsky Test (BOT-2) of motor proficiency at 6 years (*p* < 0.001) with a very strong partial eta-squared value (0.38). The strength of this statistical findings indicated a highly clinically important relationship for early motor development to proficiency at school age.

#### Child development and cognitive functioning

Another Malawi-based study revealed that child development at age five was associated with mathematical ability at age twelve (Gandhi et al., 2013). Developmental assessment at age five includes the evaluation of gross and fine motor skills, language, and socialization, and that at age twelve is based on the percentage of correct answers given to mathematics test questions, the highest grade level achieved, and the number of grade repetitions. Combining the Denver Developmental Screening Test and Griffiths Mental Developmental Scales at 5 years, the summary score was positively associated with math performance at twelve years (*p* = 0.031). Fine motor score at 5 years was also associated with the math performance (*p* = 0.011 MI), and this relationship was not modified by school attendance or grades that had to be repeated. The overall association between early development and school performance was weak, although there were moderate strength associations with math skills at twelve years.

#### Cognitive style

One study proved that a child who thinks positively about themselves, their future, and the world around them makes better educational progress (Cortina et al., 2019). This study showed that interventions that strengthen and enhance a child’s cognitive style can reduce the number of school dropouts, strengthen school performance, and improve a child’s lifelong trajectories. Having more positive cognitions was associated with progressing at least three grade levels with 7 years (adjusted OR 1.43, 95% CI 1.14–1.79), but this was not associated with emotional and behavioral problems as measured by the SDQ. The authors concluded that future studies should evaluate whether interventions to improve cognition in childhood, or to improve psycho-social adjustment (boost the SDQ score) could result in children adapting better and progressing in a more timely manner by grade level in the school setting.

#### Influence of early development on cognitive and behavioral outcomes

According to one study on early child development, the assessment of mental development can significantly predict preschool outcomes at age 5 (Hsiao & Richter, 2014). The study revealed that children with poor mental development at 1 year of age also displayed poor cognitive and behavioral development at 5 years, confirming the relationship between their development at the two ages. The relationship was significant for the Griffith’s General Quotient (*p* < 0.01) and driven primarily by Scale A: Locomotor (*p* < 0.05), Scale B: Personal-Social (*p* < 0.05), and Scale E: Performance (*p* < 0.01). However, maternal education level (completion of tertiary level 8 or not) significantly interacted with this relationship (*p* < 0.05), especially if the child was in the lower tertile (as opposed to middle or upper) at 1 year of age assessment. Maternal education level was related to higher performance at 1 year and interacted less with the relationship at 5 years, where other education-related factors were more influential.

#### Screening for at-risk children and predicting grade repetition

One study showed that psychometric assessments administered in early childhood can identify children in need of early interventions to prevent late school entry and grade repetition with moderate accuracy (Richter et al., 2015). By measuring children’s cognitive development, children at risk can be identified in a timely manner so that early interventions can be initiated during critical periods of early development (e.g., at a sensorimotor and/or per operational stage) when they can improve problem solving skills practically or conceptually (e.g., at a concreate and/or formal operational stage) neurocognitively. The Bayley Scales of Infant Development at 1 year of age and the Conners’ Teacher Rating Scale at 7 years were the most significant in predicting overall academic performance (using a receiver operating characteristic curve analysis of area beneath the curve). Other measures administered at six months and at 1 year (Griffiths Mental Development Scales, Vineland Social Maturity Scale, Behavior Screening Questionnaire) were less sensitive; as were the Denver Prescreening Developmental Questionnaire at 5 years, the Draw-a-Person task, and Raven’s Coloured Progressive Matrices at 7 years. Maternal age and low socioeconomic status were statistically predictive of Bayley performance at school entry.

#### Neurocognitive performance and children’s alertness and attention

Two of the selected studies demonstrated that the assessment of alertness and attention at 6–12 months was predictive of neurocognitive performance at 3–5 years of age (Familiar et al., 2022; Familiar-Lopez et al., 2023). Using automated eye tracking to compute proportion of time viewing a 6.2-minute animation video of colorful cartoon animals periodically moving across the screen (ECVT) at pre-school age, attention was significantly correlated with all TOVA outcomes when the child reached school age. This was especially true for the TOVA measures of vigilance attention of overall response time (*p* = 0.0128), correct response time variability (*p* = 0.0061), and ADHD index measure (*p* = 0.047)). The adjusted partial eta-squared values were moderate to strong for all three of these school-age outcomes when adjusting on the basis of caregiver training for the mother, child gender, socioeconomic status (SES), and quality of Home Observational Measurement Evaluation environment (covariate *p* = 0 0.03). None of the ECVT attention performance measures correlated significantly with any of the KABC-II cognitive ability outcomes. Using the Fagan Test of Infant Intelligence, which tracks eye gaze preference for novel human faces in our Ugandan infants at about 1 year of age, memory for unfamiliar faces (longer gaze length) and overall attention to the screen during face presentation was related to KABC-II and TOVA overall performance, especially KABC-II working memory and visual spatial-analysis measures for the children at school age.

## Discussion

This systematic review aimed to investigate the relationship between the assessment of children’s neurocognitive development and their later development. The selected studies assessed development at least twice at different ages across childhood and/or adolescence. Western-based developmental measures measuring the child’s performance such as the MSEL (Boivin et al., 2021), the Bayley Scales (Hsiao & Richter, 2014; Richter et al., 2015), and the Griffith’s scales (Richter et al., 2015), even at 1 year of age, were strongly predictive of cognitive performance at school age, also using Western-based tests [e.g., Kaufman scales (KABC-II), TOVA)]. Experimental measures of attention (ECVT) at preschool age and the Fagan Test of Infant Intelligence (working memory and attention for human faces) at 1 year of age were also predictive of KABC and/or TOVA performance at school age (Familiar et al., 2022; Familiar-Lopez et al., 2023). This stands to reason, as the ECVT and Fagan tests, when automating performance measures using eye tracking, corresponds well to MSEL overall cognitive composite performance in these Ugandan infants and toddlers (Boivin et al., 2017b; Chhaya et al., 2018).

The Bayley scales are generally considered to be the gold standard in performance-based developmental assessment (Bradley-Johnson, 2001; Drotar et al., 1999). However, the MSEL was designed to be a suitable alternative to the Bayley scales in resource-constrained setting where a high level of psychometric assessment expertise was not available (Kammerer et al., 2013; Mullen, 1995). Overall cognitive and motor performance on the Mullen and the Bayley scales are highly correlated in Western-based samples of children (Bradley-Johnson, 2001). Screening tests that are not performance based but instead are largely based on parental report (e.g., Denver Developmental Screening Tests, Ages and Stages Questionnaire, SDQ) are less predictive of cognitive, academic achievement, and psychosocial adjustment outcomes at school age (Cortina et al., 2019; Zuilkowski et al., 2016).

In a study of Ugandan children perinatally HIV infected, exposed and uninfected (HEU), and not exposed or infected (HUU), Bayley cognitive and motor performance measures identified developmental delays in the HIV-positive cohort when assessed at 6, 12, 18, and 24 months (Drotar et al., 1997; Drotar et al., 1999). The Fagan Test of Infant Intelligence (without automatic eye-tracking measures) did not. When these same cohorts were assessed again at school-age with the KABC, overall Bayley cognitive performance from early childhood was generally predictive of KABC mental processing ability, especially for the Simultaneous Processing domain (visual-spatial analysis and problem solving subtests) (Bagenda et al., 2006). Again, Western-based performance based developmental assessments were predictive of western-based cognitive ability tests when appropriately adapted to the sub-Saharan context with well-matched comparison groups (e.g., HIV, HEU, and HUU cohorts longitudinally assessed).

Some studies have indicated that neuropsychological assessment in childhood is strongly predictive of children’s later cognitive development (Boivin et al., 2021; Familiar et al., 2022; Familiar-Lopez et al., 2023). According to the first identified study, early childhood neurodevelopmental assessment predicts the cognitive and neuropsychological abilities of children in Benin (Boivin et al., 2021). In another study assessing the neurocognitive and behavioral development of Malawian children suffering from severe malaria, the results indicated that early childhood developmental assessment can predict their neuropsychological performance in later years (Boivin et al., 2019a). These findings show that in both healthy and malaria-affected children, developmental measures can predict later neurocognitive outcomes. It is important to note when comparing the Benin to the Malawi follow-up assessments that both the KABC-II and TOVA were used for the assessment of school-age cognitive performance. For the preschool performance-based developmental assessment, however, the western-based MSEL was administered at 1 year of age for the Benin cohort, while the MDAT—developed, validated, and normed for Malawian children—was used for the malaria cohorts. Irrespective of whether the Western-based MSEL or the Malawian-based MDAT was used, the predictive correspondence for KABC-II overall cognitive ability was very significant with comparable effect sizes especially on the language development scales (Boivin et al., 2019a, 2021).

Similarly, studies on children’s alertness and attention have shown that eye-tracking-based measurements of infant attention and working memory can predict some features of neurocognitive performance (Boivin et al., 2017b; Chhaya et al., 2018). Such studies benefit the population in terms of overall health and allow for the early support and care of children unable to achieve their cognitive developmental potential in the sub-Sahara (Ajayi et al., 2017). In terms of schooling, this review identified studies that used neurodevelopmental assessment to predict academic and educational development and progress in the sub-Sahara (Bangirana et al., 2011a,b). This study showed that children’s development at age 5 was associated with mathematical abilities at age twelve (Gandhi et al., 2013). These results highlight the crucial role of early normal development on children’s academic achievement (Ribe et al., 2018).

The review also identified data on children’s mental development and perception of themselves, their environment, and their future and the impact of this perception on their educational progress. The south African study showed that positive perceptions in children undoubtedly lead them to achieve better educational progress (Cortina et al., 2019). Thus, children’s cognitive style predicts their academic educational progress; considering the neurocognitive evaluation of children in health and educational institutions in the sub-Sahara would, therefore, be useful. This would promote human development by offering children an opportunity to take full advantage of their potential and reduce the number of school dropouts.

In a similar vein, another study found that social and environmental adversities in childhood and externalizing disorders are associated with an increased risk of suicidal ideation in adolescence and young adulthood (Orri et al., 2022). This study revealed how individual, family, and environmental factors in childhood are likely to harm an individual’s future (Familiar et al., 2019; Familiar et al., 2020). Additionally, in investigating the influence of early development on preschool cognitive and behavioral outcomes, one study showed that mental development assessed at 1 year of age significantly predicts preschool outcomes at age five; the children with poor mental development at 1 year of age displayed the lowest cognitive and behavioral development at age five (Hsiao & Richter, 2014). However, this study also found that higher levels of maternal education could mitigate the negative impacts of early developmental delay on children’s preschool cognitive and behavioral outcomes. Low maternal education is a major risk factor for lower intelligence quotients (IQs) in preschool children (Charkaluk et al., 2017). Thus, maternal education levels are also a major predictor of cognitive outcomes for children in sub-Sahara (Boivin et al., 1996). The current review highlights the link between psychometric assessment administered in early childhood and the screening and identification of those in need of early interventions (Richter et al., 2017). It demonstrates that the early identification and screening of at-risk children allows one to anticipate their care needs to avoid grade repetitions or delayed school entry (Richter et al., 2015).

Finally, this review reveals that assessments using neurocognitive tests designed and created in developed countries but used in different cultural and socioeconomic contexts in SSA also produce significant and non-negligible results in the assessment of child neurocognitive development (Bodeau-Livinec et al., 2019). Moreover, it would be timely to develop appropriate tests with concordant ecological and cultural validity for countries in sub-Sahara (Gladstone et al., 2014; Kammerer et al., 2013; Semrud-Clikeman et al., 2017). In recent years, researchers have devoted much effort to studying the neurocognitive development of children in developing countries, with increasing interest in neurocognitive assessments (Black et al., 2017, 2023; Grantham-McGregor & Walker, 2023).

## Limitations

Although the results of this review are encouraging for the assessment of child neurocognitive development in the sub-Sahara, there are several limitations related to data collection that merit discussion. The protocols of the selected studies were not evaluated with respect to their methodologies. Therefore, one can question the validity of the tests used in these studies, the contexts in which they were used, the different statistical strategies for standardizing test scores, and the significance of the results of these assessments in the cultural and SES contexts of developing countries. Also, none of the studies included predicted performance beyond 12 years of age. This is a limitation of our systematic review, limiting the generalizability of our conclusions beyond this age.

Nevertheless, the main findings are promising in terms of the use of neurodevelopmental assessments in infancy or childhood. This is because even when using HIC-based developmental assessments in early childhood, outcomes from such measures were predictive of children’s later cognitive and neuropsychological abilities. This included the ability of HIC-based early childhood developmental assessments to predict school-age cognitive functioning, cognitive styles and later mental health, and likelihood for grade repetition or delayed school entry.

## Conclusion

This systematic review assessed the quality and synthesized the main findings of studies measuring cognitive development in cohorts of Sub-Saharan African children at a minimum of two different ages to assess whether it is associated with children’s later cognitive development and mental health. In this synthesis, we found that researchers used a variety of neuropsychological tests to assess neurocognitive development in SSA children, and that these assessments are predictive of children’s neurocognitive development in later years. We also noted that longitudinal studies are increasingly being used in research to obtain more adequate long-term follow-ups of children in this setting with multiple and varied risk factors. This review opens avenues for further research and may lead to the exploration of studies assessing children using tests created from and employing local materials and then validated according to the SES and cultural contexts in SSA.

**Fig. 2:**
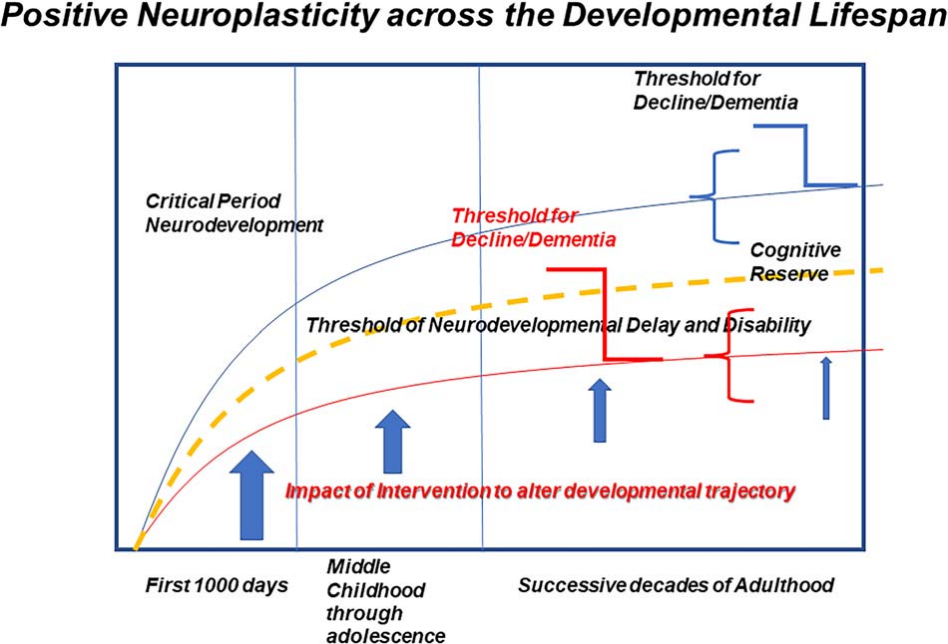
Dramatic progression of development in the first 1000 days (including gestation) is depicted for normal (upper trajectory) and delayed (lower line) children. The imperative impact of intervention for at-risk children is also depicted with vertical vectors during the first 1000 days (greatest impact), middle childhood (moderate impact), and throughout successive decades of adulthood (diminishing impact). The compensatory range for neurodevelopmental function available in adulthood is pictured to the right side of this figure, the reach for which is a function of the trajectory in the first 1000 days.

Future directions of brain/behavior assessment research which strengthens the ability of measures in early child development to predict later neuropsychological function at school age and beyond is foundational to the potential impact and importance of this work (Fig. 2). Figure 2 illustrates one such application. In this figure, the dramatic progression of development in the first 1000 days (including gestation) is depicted for normal (upper trajectory) and delayed (lower line) children. The imperative impact of intervention for at-risk children is also depicted with vertical vectors during the first 1000 days (greatest impact), middle childhood (moderate impact), and throughout successive decades of adulthood (diminishing impact). The compensatory range for neurodevelopmental function available in adulthood is pictured to the right side of this figure, the reach for which is a function of the trajectory in the first 1000 days.

Performance-based developmental assessment in the first 1000 days with such Western-based measures as the BSID, the MSEL, or the MDAT are important in identifying children with developmental delays who could benefit most from interventions to minimize risk and maximize resilience when provided in the first 1000 days; during gestation and after through infancy and in very early childhood. Such evidence-based interventions have been systematically reviewed and proposed for scale-up as an integral part of well-child care in the first 1000 days as part of a seminal Lancet launch in 2017 (Black et al., 2017; Britto et al., 2017; Daelmans et al., 2017; Lo et al., 2017; Richter et al., 2017; Shawar & Shiffman, 2017). More recently, *Lancet* has launched a series on the strategic importance of training caregivers in LMICs to provide emotionally responsive and nurturing caregiving to their infants and toddlers (Black et al., 2023).

Validated and culturally appropriate tests are then needed to gauge the benefits of such early child development caregiver training interventions throughout early and into middle childhood (Gentchev et al., 2022). In fact, the importance of early-life risk factor identification for the early intervention in the remediation of neurocognitive disabilities throughout childhood and into adulthood has recently been substantiated in a longitudinal study of four SSA cohorts by Stein and colleagues in South Africa (Stein et al., 2023). This is the future direction of this type of assessment research, particularly in LMICs, as applied in the first 1000 days to minimize developmental risk and maximize resilience for evidence-based interventions.

Although based mostly on studies with African children, the present systematic review provides evidence in support of how early developmental assessment can predict cognitive performance at a later time for at-risk children in LMICs globally (Boivin & Giordani, 2013). In future studies, at-risk cohorts can be longitudinally followed through childhood and into adolescence and early adulthood. Doing so will allow us to monitor the impact of early interventions on the children’s overall developmental trajectory in later years, identifying those early interventions most strategic in the neurocognitive support of highly at-risk children over the long term. Such work can also identify interventions for teens and adolescents that are appropriate and effective in remediating persisting neuropsychological and psychosocial difficulties, as well as how best to screen for the emergence of such pathologies at earlier stages in a child’s development.

## Funding

Fondation de France, France (00100075).

## Disclosure statement

Authors declare no conflict of interest.

## Author Contributions

Romeo Zoumenou (Conceptualization, Formal analysis, Investigation, Methodology, Visualization, Writing—original draft), Florence Bodeau-Livinec (Conceptualization, Formal analysis, Funding acquisition, Investigation, Methodology, Project administration, Writing—review and editing), Léa Chausseboeuf (Data curation, Formal analysis, Methodology, Writing—review and editing), Michael Boivin (Investigation, Writing—review and editing), Jaqueline Wendland (Investigation, Methodology, Writing—review and editing).
